# Laryngeal Phthisis

**Published:** 1873-11

**Authors:** Morell Mackenzie

**Affiliations:** London


*MEDICAL RECORD:*

LARYNGEAL PHTHISIS.—*Morell Mackenzie, M.D., London.*—The patients frequently speak of having caught cold not long ago. No doubt in these, as in most other cases, the origin of actual trouble in the larynx is to be found in catarrhal affections of the mucous membrane covering the respiratory tract. It is important in this place to observe, there is very little expectoration in diseases of the larynx. Upon this symptom the differential diagnosis with lung affections may be based in part. If there is much expectoration in a given case, we may be positive that the lungs are attacked, whether it be by bronchitis, tubercle, or still some other form of lung trouble. In laryngeal phthisis dysphagia is frequently owing to the fact that the epiglottic valve, in the effort of deglutition, imperfectly closes the laryngeal opening, and food passes into its cavity. The patients, after vain endeavors to accomplish this function, refuse to eat except so far as is rendered imperative by a feeling of intense hunger. Soon the loss of flesh and strength follow.

The way to meet this difficulty is to introduce a tube through the mouth, just below the epiglottis, and administer nutritive substances by means of injections made by the tube. There is no necessity to pass the tube down so far as the stomach. The aliments must not always be the same. They are to be varied constantly, and the diet should not be made up exclusively of finely divided meat and soups. Vegetable food ought also to be given from time to time, and in the form of purl of different kinds.

In all forms of laryngeal phthisis, we may consider it as settled that little really useful can be done outside of local applications. Amongst these Dr. Mackenzie especially recommends the use of the solution of perchloride of iron (in the proportion of a drachm of solid ferri-perchlorid to an ounce of water). This solution gives much temporary relief, and is especially serviceable in the form where pyriform tumefaction exists.

In some cases where the iron is no longer to be depended upon for giving even temporary good results, we may resort with considerable advantage to the use of solutions of the chloride of zinc. According to Marcet, scarifications, followed by applications of tincture of iodine, may be relied upon. Mackenzie affirms that he has sometimes found bad consequences to follow this method of treatment, and as we are unable to cure the disease, it is better and safer to confine our local treatment of laryngeal phthisis to the employment of the astringent palliatives.

